# Supplementary material for: Cep55 promotes cytokinesis of neural progenitors but is dispensable for most mammalian cell divisions
Source: Nat Commun. 2020 Apr 8;11:1746. doi: 10.1038/s41467-020-15359-w (PMC7142149; doi:10.1038/s41467-020-15359-w)
Supplement: Supplementary file 1 — Supplementary Information [file 41467_2020_15359_MOESM1_ESM.pdf]

Supplementary Information

Cep55 promotes cytokinesis of neural progenitors but is dispensable for most mammalian cell divisions.

Tedeschi et al.

This PDF includes:

Supplementary Figures 1 to 17

Supplementary references

Supplementary Figure 1

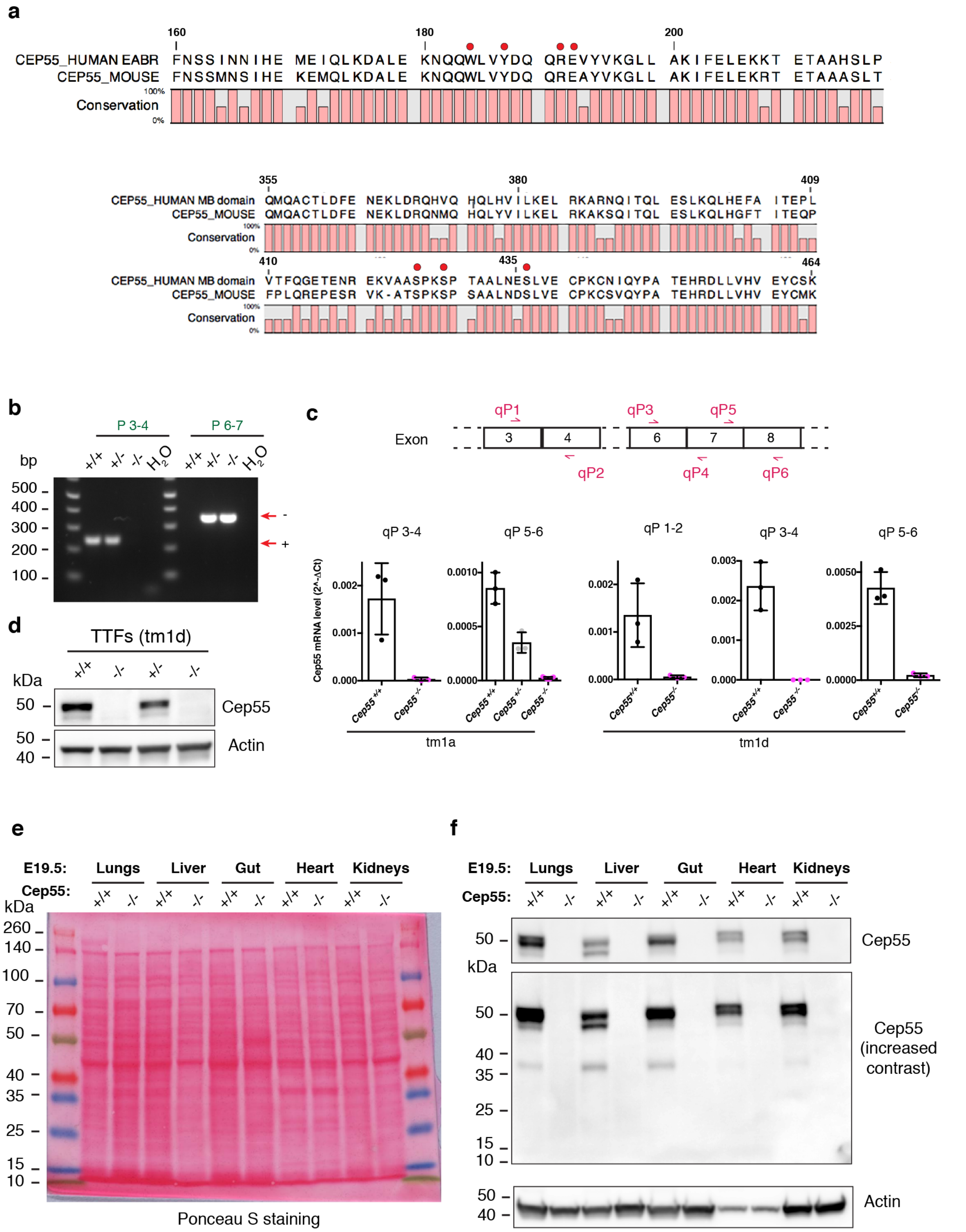

Supplementary Figure 1: **Validation of *Cep55* targeting strategy.** (**a**) Schematic showing alignment of human and mouse Cep55 domains. Human EABR as identified in (Leet et al 2008) and MB domain as identified in (Fabbro et al 2006). Red dots indicate key functional residues (Lee et al 2008; Fabbro et al 2006). (**b**) PCR analysis of primary tail fibroblasts with primers P3, 4, 6 and 7 shown in Fig.1a to verify *Cep55* status. (**c**) Quantitative reverse

transcription PCR (qRT–PCR) of relative Cep55 mRNA levels in fibroblasts of the indicated genotypes. Results were normalized to Gapdh. Means ± SD are shown; n = 3 independent experimental repeats for each genotype, except for tm1a-*Cep55*$^{-/-}$ qP 5-6 (n=7). (**d**) Western blots of protein extracts from mouse tail tip primary fibroblasts (TTFs) of the indicated genotypes with antibodies against Cep55 and Actin; n= 2 independent experiments (**e**) Ponceau S staining of protein extracts from the indicated embryonic E19.5 tissues and genotypes (*Cep55* tm1a mice); n= 1 independent experiment. (**f**) Western blots of protein extracts from (e) with antibodies against Cep55 and Actin; n= 1 independent experiment.

Supplementary Figure 2

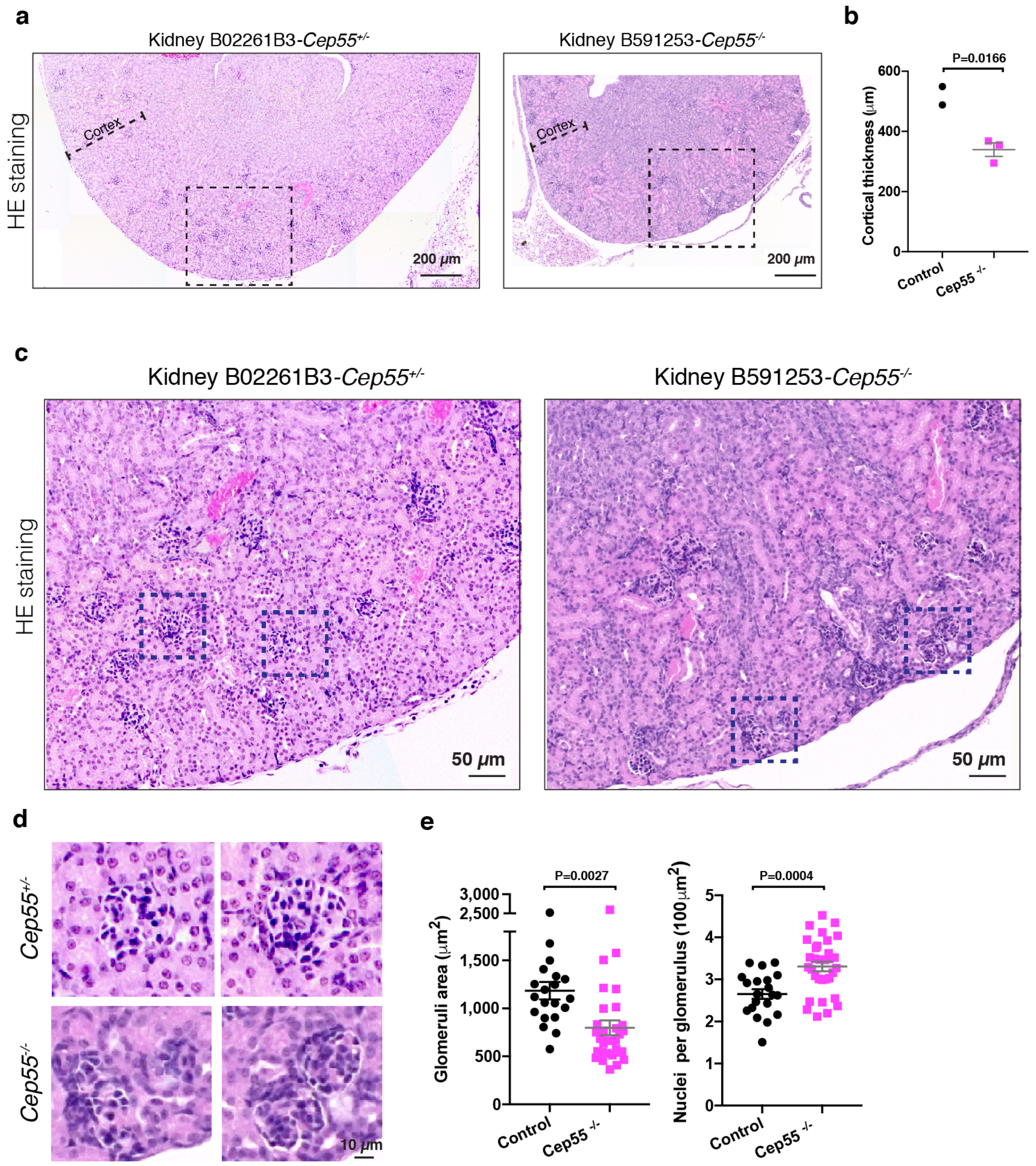

Supplementary Figure 2: **Kidney dysmaturity in *Cep55* knockout mice.** (**a-c**) HE staining (a,c) and cortical thickness measurements (b) of kidneys of two-week-old mice of the indicated genotypes. Horizontal bars indicate mean; error bars indicate SEM. n= 2 and 3 mice, respectively; *P* values calculated using Student's two-tailed unpaired t test. Dotted boxes in (a) and (c) indicate areas enlarged in (c) and (d) respectively. (**d**) Enlarged view of glomeruli from (c). (**e**) Quantification of glomeruli area (left) and nuclei per glomerulus (right). Horizontal bars indicate mean; error bars indicate SEM. n = 2 and 3 mice, respectively; 20 and 32 glomeruli analysed, respectively; *P* values calculated using Student's two-tailed unpaired t test.

Supplementary Figure 3

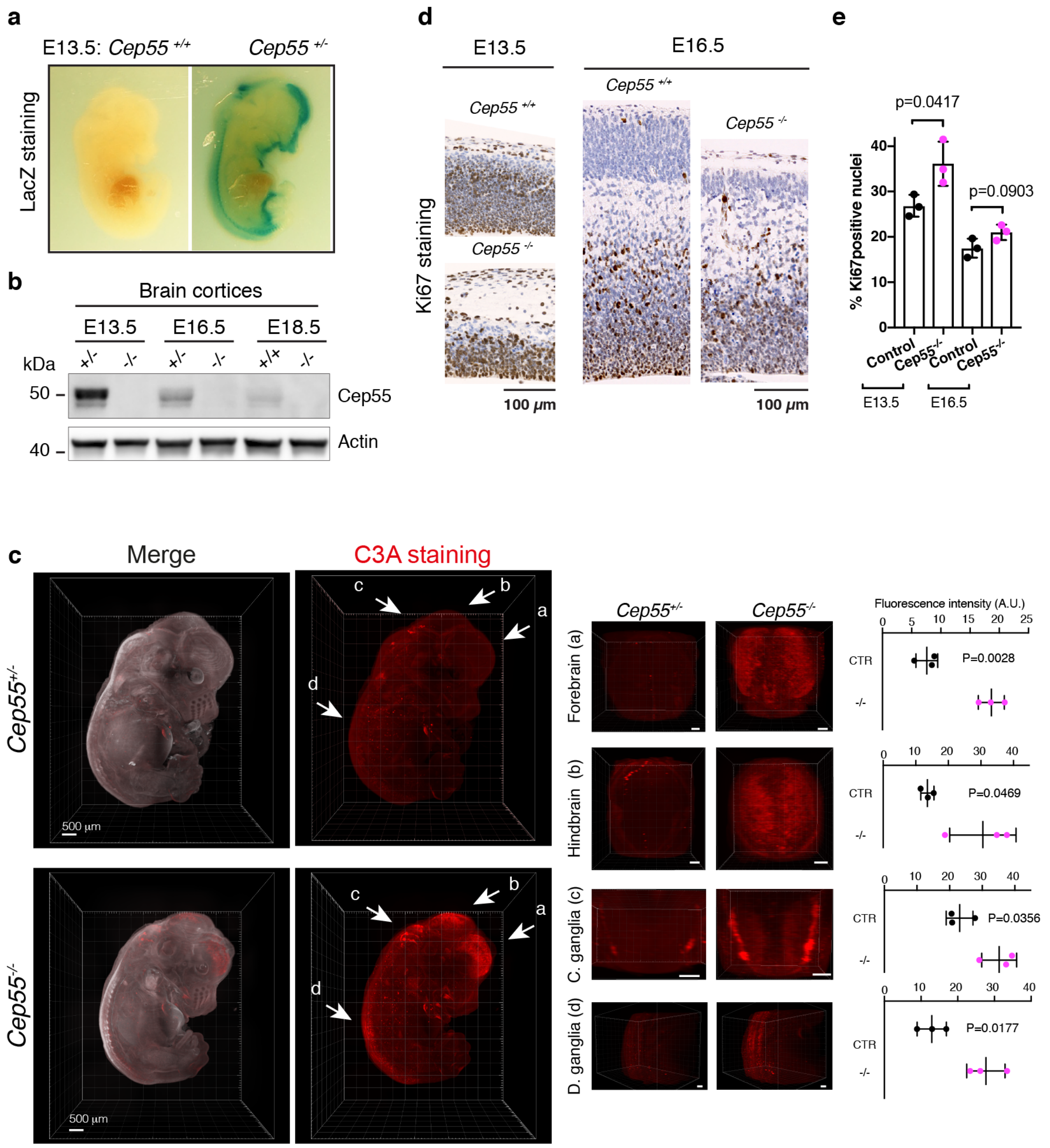

Supplementary Figure 3: **Characterization of *Cep55* knockout brain phenotype.** (**a**) LacZ staining in E13.5 *Cep55*$^{+/+}$ and *Cep55*$^{+/-}$ embryos; n=1 independent experiment. (**b**) Western blots of protein extracts from brain cortices of mice of the indicated genotypes and developmental stages with antibodies against Cep55 and Actin; n= 2 independent experiments (**c**) FLASH visualization (3D view) of whole E13.5 embryos of the indicated genotypes stained for active caspase 3 (C3A). The grey signal in the merge is auto-fluorescence from the 488 channel, used for visualising the outline of the embryo. C3A signal from the indicated areas (white arrows) is quantified on the right. A.U., arbitrary units. Scale bars, 200 µm. *P* values calculated using Student's two-tailed unpaired t test. Graphs show mean ± SD. (**d**) Immunohistochemistry of comparable regions of *Cep55*$^{+/+}$ and *Cep55*$^{-/-}$ mouse cortices, at the indicated stages, for the proliferation marker Ki67; n= 3 independent experiments. (**e**) Quantification of Ki67-positive (Ki67+) nuclei from c. Control includes *Cep55*$^{+/+}$ and *Cep55*$^{+/-}$ mice. n = 3 mice per genotype and per stage; cell numbers quantified for control and *Cep55*$^{-/-}$ respectively were 2248 and 1289 (E13.5), 3470 and 2237 (E16.5). *P* values calculated using Student's two-tailed unpaired t test. Bar charts show mean ± SD.

Supplementary Figure 4

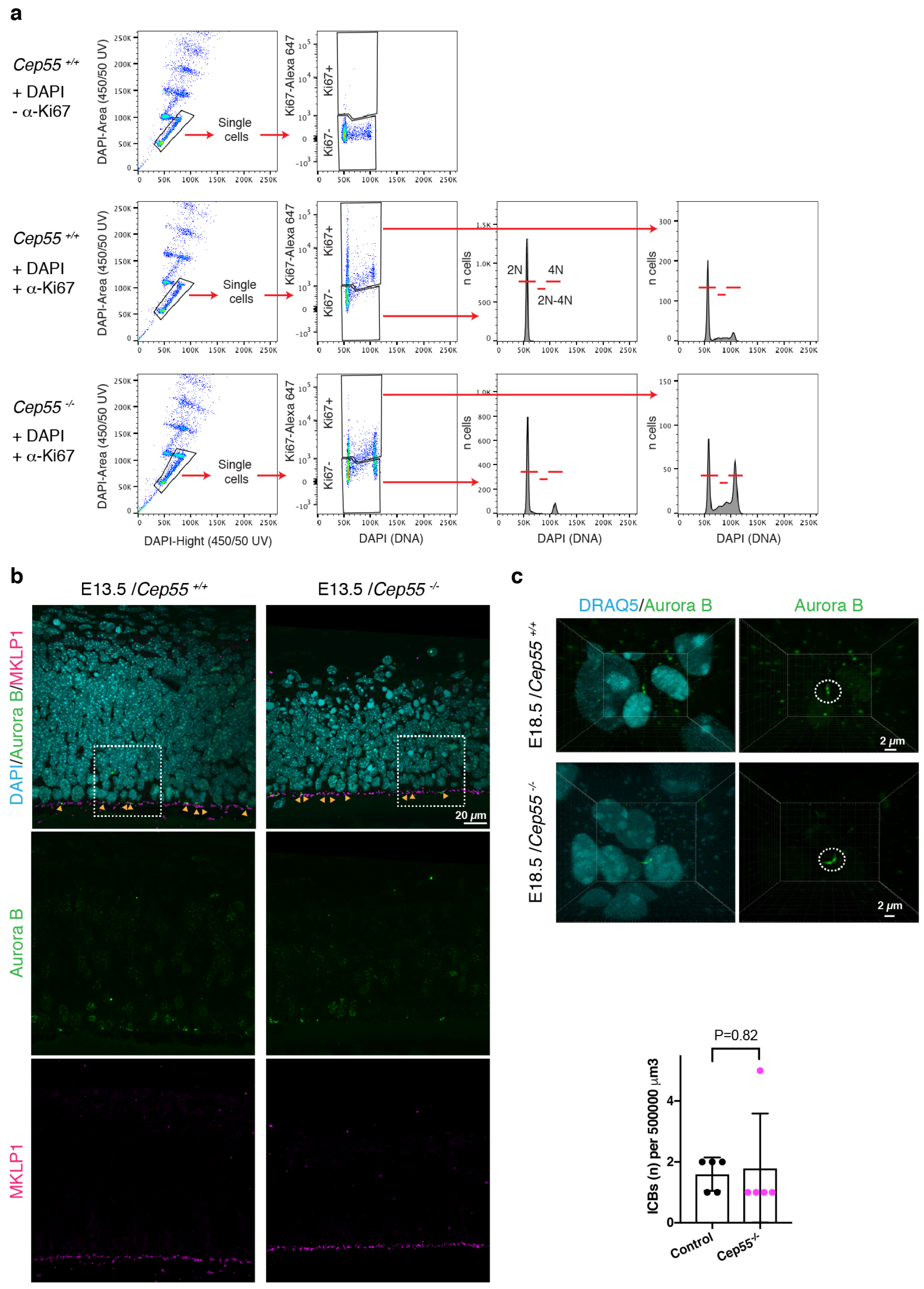

Supplementary Figure 4: **The number of intercellular bridges in the brain cortex is similar in *Cep55*$^{+/+}$ and *Cep55*$^{-/-}$ embryos.** (**a**) Gating strategy for the cell cycle analysis shown in Figure 4a-c. (**b**) Confocal 3D images of E13.5 cortex from brains of the indicated genotypes stained with DAPI (DNA), Aurora B (green) and MKLP1 (Magenta). Boxed areas are shown in Fig. 4h. Arrowheads indicate intercellular bridges positive for Aurora B and MKLP1, as shown in the enlarged images in Fig. 4h. (**c**) FLASH imaging (3D view) of E18.5 cortices stained with DRAQ5 (DNA) and an antibody against Aurora B (green). (**c**) Quantification of Aurora B-positive intercellular bridges (ICBs) as shown in (b). Bar charts show mean ± SD. n = 5 mice per genotype; n = 8 and 9 ICBs, respectively. *P* values calculated using Student's two-tailed unpaired t test.

Supplementary Figure 5

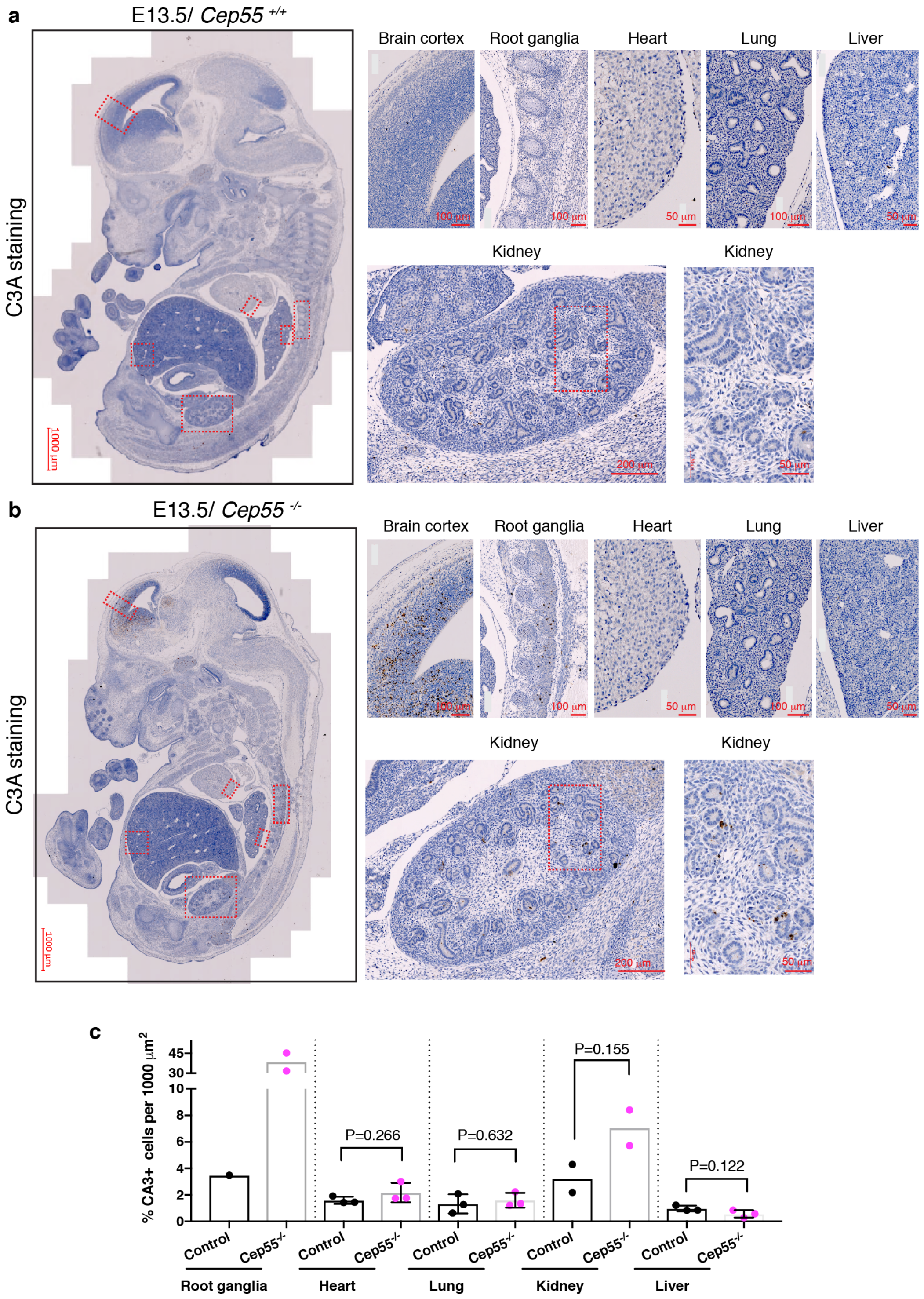

Supplementary Figure 5: **Apoptosis is increased in the spinal cord and kidney of *Cep55*$^{-/-}$ embryos.** (**a** and **b**) Immunohistochemical staining of *Cep55*$^{+/+}$ and *Cep55*$^{-/-}$ E13.5 embryos for active Caspase 3 (C3A). (**c**) Quantification of C3A-positive (C3A+) cells (brown stain) from (a) and (b) in the indicated organs using QuPath (Methods). Bar charts show mean ± SD. n = 1, 2, 3, 3, 3, 3, 2, 2, 3 and 3 mice per genotype; n = 2108, 4508, 14318, 11013, 6961, 17893, 8124, 13685, 6718 and 12286 cells quantified, respectively. *P* values calculated using Student's two-tailed unpaired t test.

Supplementary Figure 6

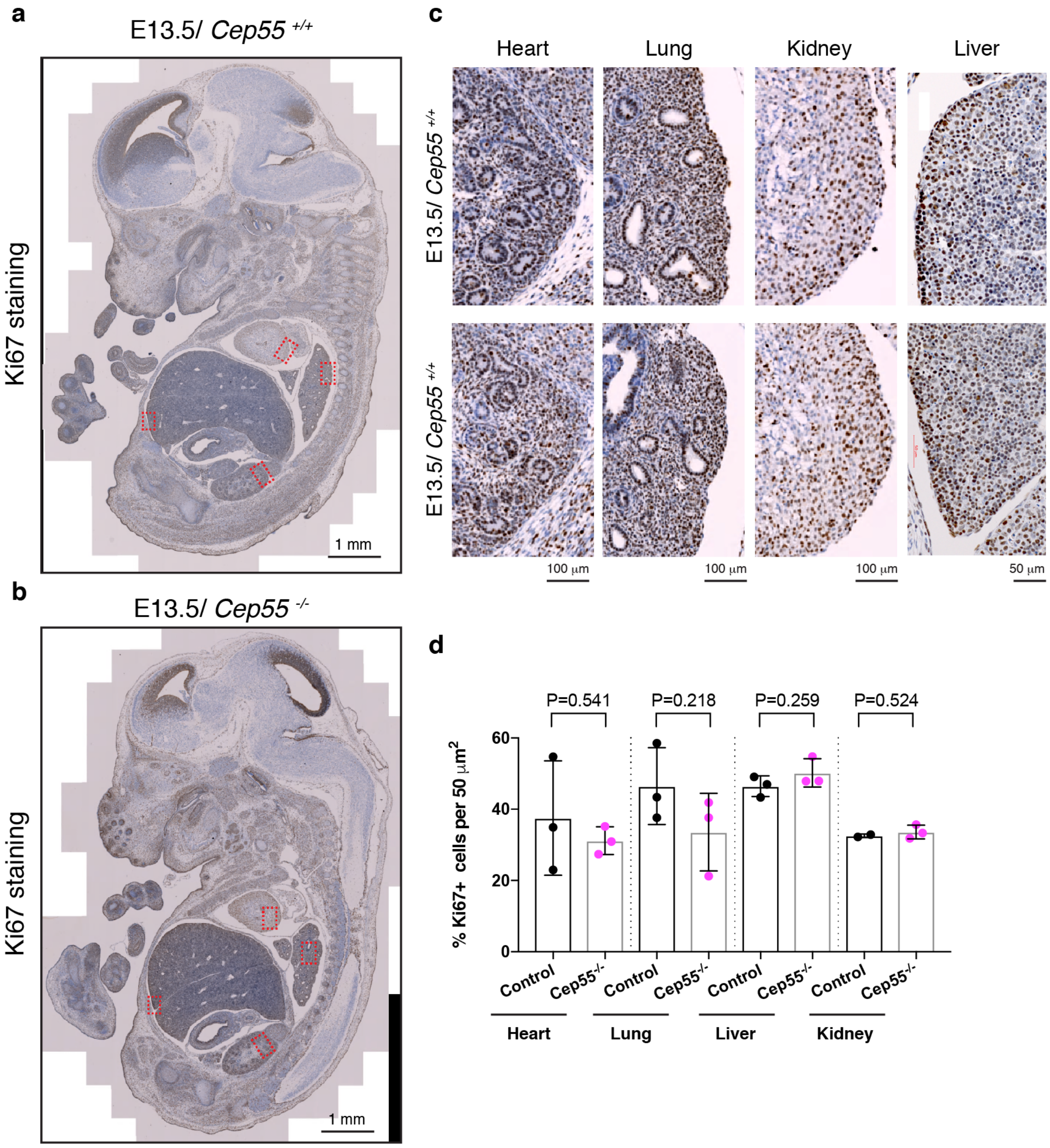

Supplementary Figure 6: **Proliferation rates are similar in *Cep55*$^{+/+}$ and *Cep55*$^{-/-}$ embryos.** (**a** and **b**) Immunohistochemical staining of *Cep55*$^{+/+}$ and *Cep55*$^{-/-}$ E13.5 embryos for Ki67. (**c**) Quantification of Ki67-positive (Ki67+) cells (brown stain) from (a) and (b) in the indicated organs using QuPath (Methods). Bar charts show mean ± SD. n = 3 mice per genotype; n = 9835, 13696, 12218, 14124, 10555, 13881, 8101 and 154328 cells quantified, respectively. *P* values calculated using Student's two-tailed unpaired t test.

Supplementary Figure 7

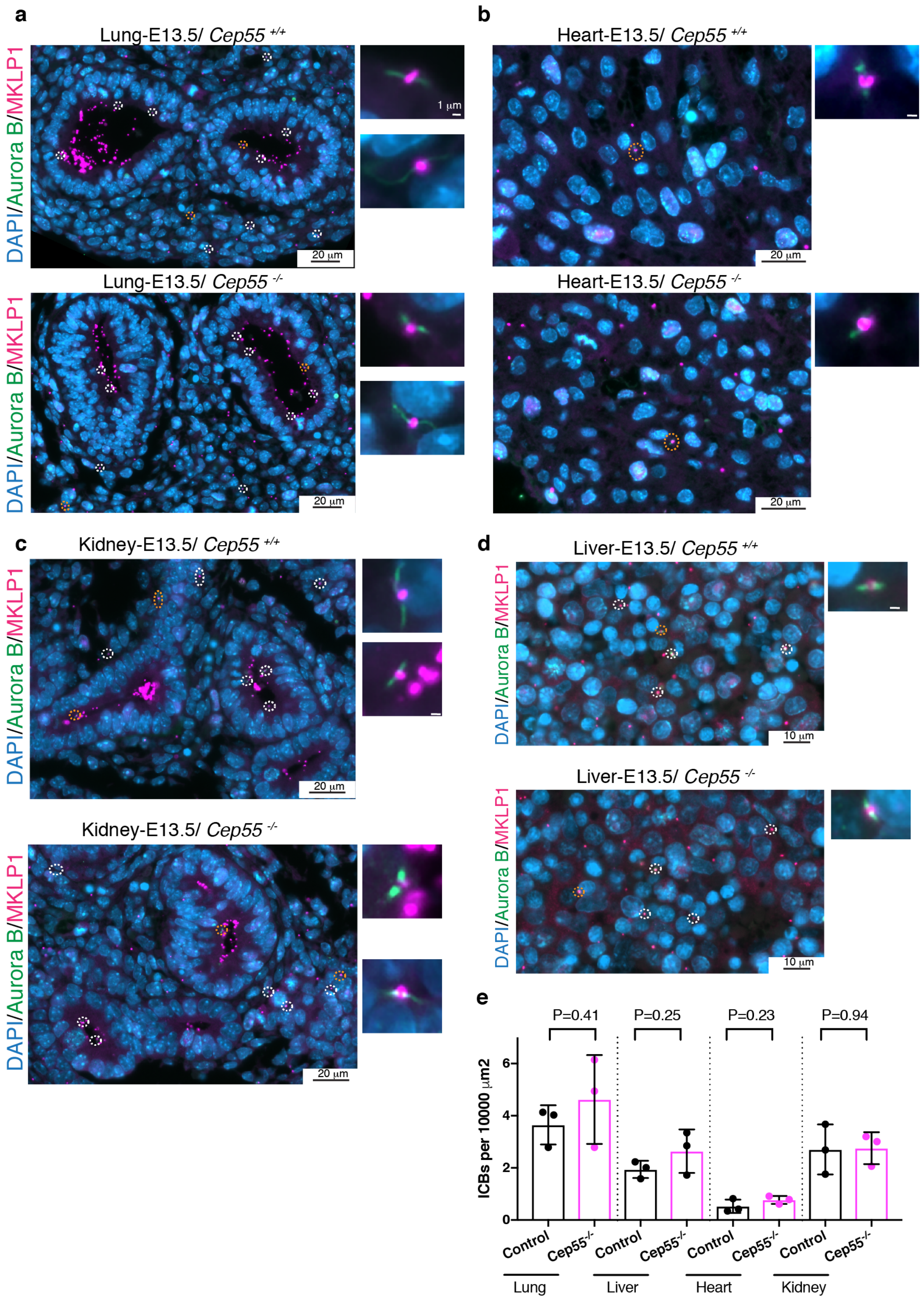

Supplementary Figure 7: **The number of intercellular bridges in the lung, liver, heart and kidney is similar in *Cep55*$^{+/+}$ and *Cep55*$^{-/-}$ embryos.** (**a-d**) Sections of E13.5 mouse organs of the indicated genotypes were stained with DAPI (blue), Aurora B (green) and MKLP1 (magenta). Dotted lines indicate ICBs. Areas outlined in yellow are shown on the right of the corresponding organ (a, lung; b, heart; c, kidney; d, liver). (**e**) Quantification of intercellular bridges (ICBs) as shown in (a)-(d). Bar charts show mean ± SD. n = 3 mice per genotype; n = 125, 106, 71, 94,70, 88, 139 and 119 ICBs, respectively. *P* values calculated using Student's two-tailed unpaired t test.

Supplementary Figure 8

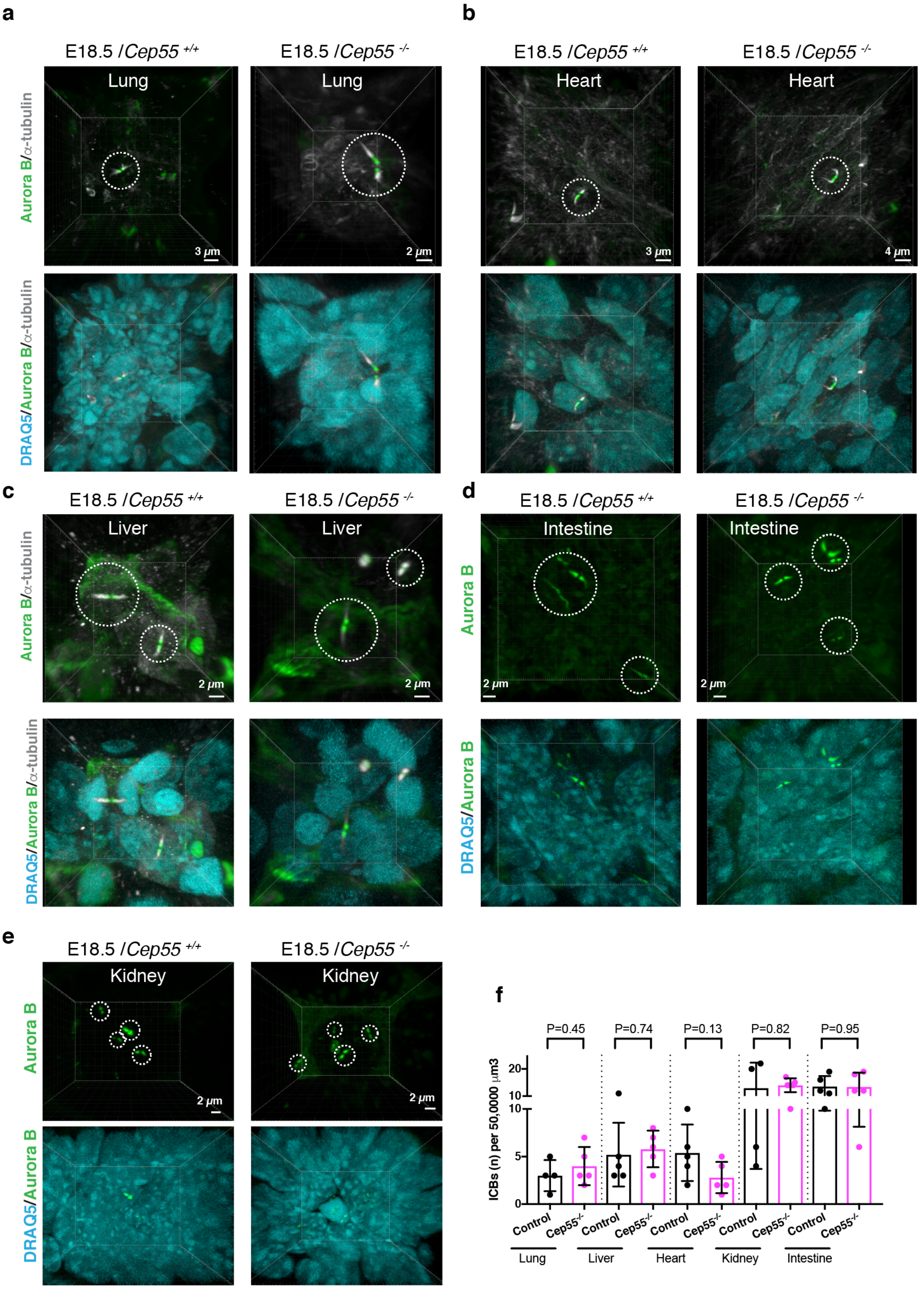

Supplementary Figure 8: **The number of intercellular bridges in the lung, liver, heart, intestine and kidney is similar in** ***Cep55***$^{+/+}$ **and** ***Cep55***$^{-/-}$ **embryos, as shown by FLASH imaging.** (**a-e**) E18.5 3D tissue samples of the indicated genotypes were stained with DRAQ5 (blue), Aurora B (green) and α-tubulin (white). Dotted circles highlight intercellular bridges (ICBs). (**f**) Quantification of ICBs as shown in (a)-(e). Bar charts show mean ± SD. n = 5 mice per genotype, except lung control (4) and kidney control (4); n = 12, 20, 26, 29, 27, 14, 52, 70, 68 and 67 ICBs, respectively. *P* values calculated using Student's two-tailed unpaired t test.

Supplementary Figure 9

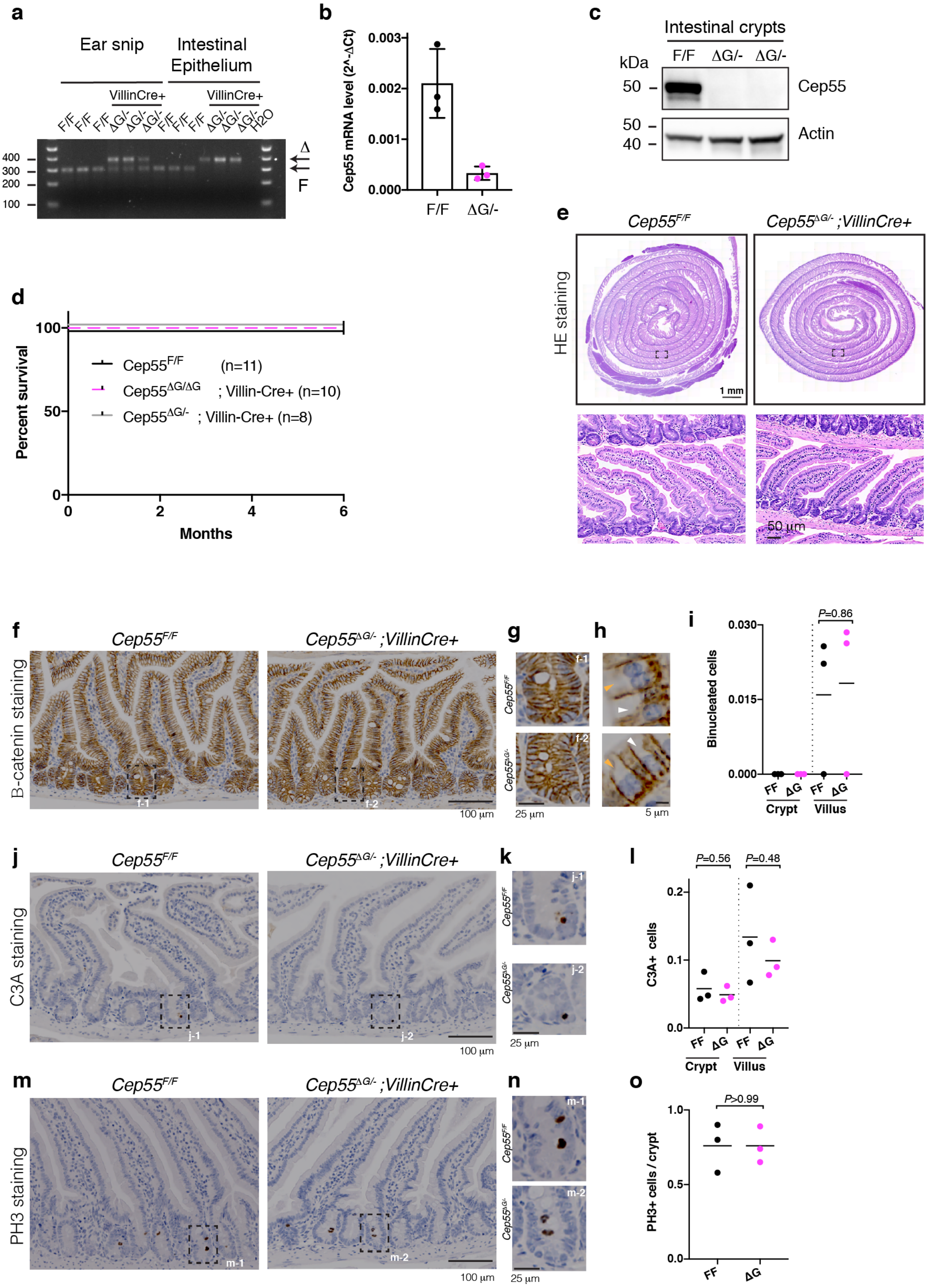

Supplementary Figure 9: ***Cep55* is dispensable in the adult mouse small intestine.** (**a**) PCR showing deletion of *Cep55* in the intestinal epithelium of 3-month-old $Cep55^{F/F}$ and $Cep55^{\Delta G/-}$; *Villin-Cre* mice using primers P3, P4, P6 and P7 (Fig. 1a). F, $Cep55^{F}$ allele; -, $Cep55^{tm1d}$ allele, ΔG, $Cep55^{tm1d}$ in the gut. Note that the band corresponding to the F allele disappears only in the intestinal epithelium of Villin-Cre+ mice. (**b**) qRT–PCR of *Cep55* mRNA levels in cells isolated from the intestinal epithelium of $Cep55^{F/F}$ (F/F) and $Cep55^{\Delta G/-}$; *Villin-Cre* mice (ΔG/-). Results were normalized to Gapdh. Bar chart shows mean ± SD. n = 3 mice per genotype. (**c**) Western blots of protein extracts from intestinal crypts of the indicated genotypes and antibodies. (**d**) Kaplan-Meier curves showing survival of mice of the indicated genotypes. P>0.9999 for F/F vs ΔG/- and F/F vs ΔG/ΔG. *P* values calculated using Mantel-Cox test. (**e**) HE staining on representative small intestines of $Cep55^{F/F}$ and $Cep55^{\Delta G/-}$; *Villin-Cre* mice. Boxed areas are magnified below. **(f)** Immunohistochemistry of adult small intestine from mice of the indicated genotypes for Β-catenin. Boxed areas, which contain the proliferative intestinal crypts, are magnified in (**g**). (**h**) Magnified areas of intestinal villi containing binucleated cells (yellow arrowhead) and mononucleated cells (white arrowhead). (**i**) Quantification of binucleated cells in crypts and villi as shown in (g and h). Horizontal bars indicate mean. n = 3 mice per genotype; 89 and 82 crypts, 43 and 37 villi per genotype were analysed. (**j**) Immunohistochemistry of adult small intestine for active caspase 3 (C3A). Boxed areas are magnified in (**k**). (**l**) Quantification of C3A-positive (C3A+) nuclei in crypts and villi as shown in (j and k). Horizontal bars indicate mean. n = 3 mice per genotype; 68 and 63 crypts, 45 and 39 villi per genotype were analysed. (**m**) Immunohistochemistry of adult small intestine for phospho-histone 3 (PH3). Boxed areas are magnified in (**n**). (**o**) Quantification of PH3-positive (PH3+) cells in crypts as shown in (n). Horizontal bars indicate mean. n = 3 mice per genotype; 30 and 29 crypts were analysed, respectively. *P* values in (i), (l) and (o) calculated using Student's two-tailed unpaired t test.

Supplementary Figure 10

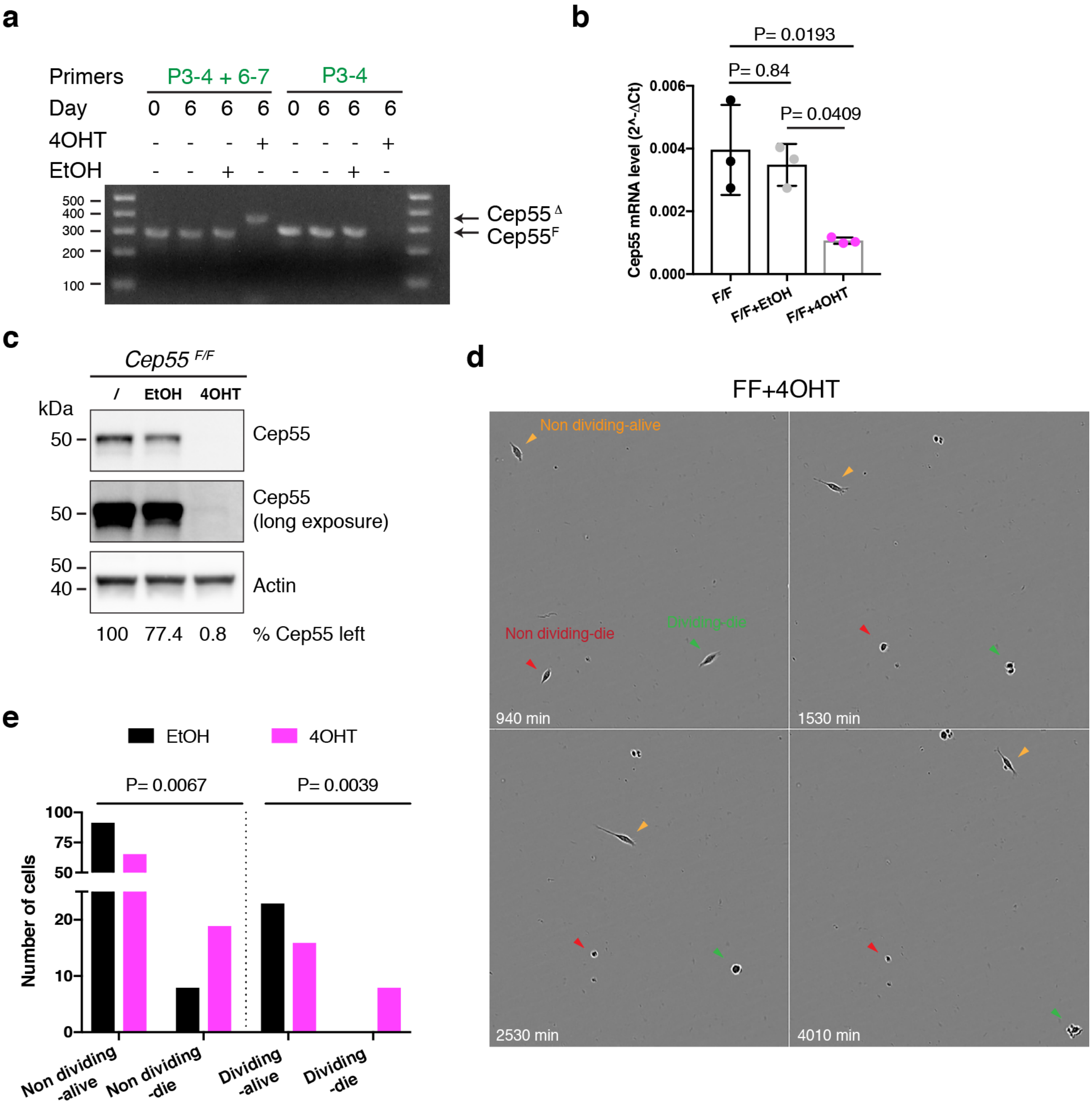

Supplementary Figure 10: **Characterization of neural progenitors depleted for Cep55 *in vitro*.** *Cep55* was deleted in *Cep55*$^{F/F}$; *R26*$^{CreERT2/+}$ NPCs as indicated in Fig. 5a. (**a**) PCR analysis with the indicated primers shows *Cep55* deletion 6 days after addition of 4OHT. 4OHT, 4-hydroxytamoxifen; EtOH, ethanol. (**b**) qRT–PCR of relative Cep55 mRNA levels 6 days after the indicated treatments. Results were normalized to Gapdh. Bar chart shows mean ± SD (n=3 independent experiments). * P values were calculated by one way ANOVA followed by Tukey's multiple comparisons test (**c**) Western blots of protein extracts from day 6 with antibodies against Cep55 and Actin; n=1 experiment. (d) Time-lapse images showing examples of dividing and non-dividing Cep55-deleted NPCs that either remain alive or die during a 3-day time course. Events are quantified accordingly in (e). n= 123 control and 109 *Cep55* deleted total cells, respectively. P values were calculated by Fisher's exact test (two-sided).

Supplementary Figure 11

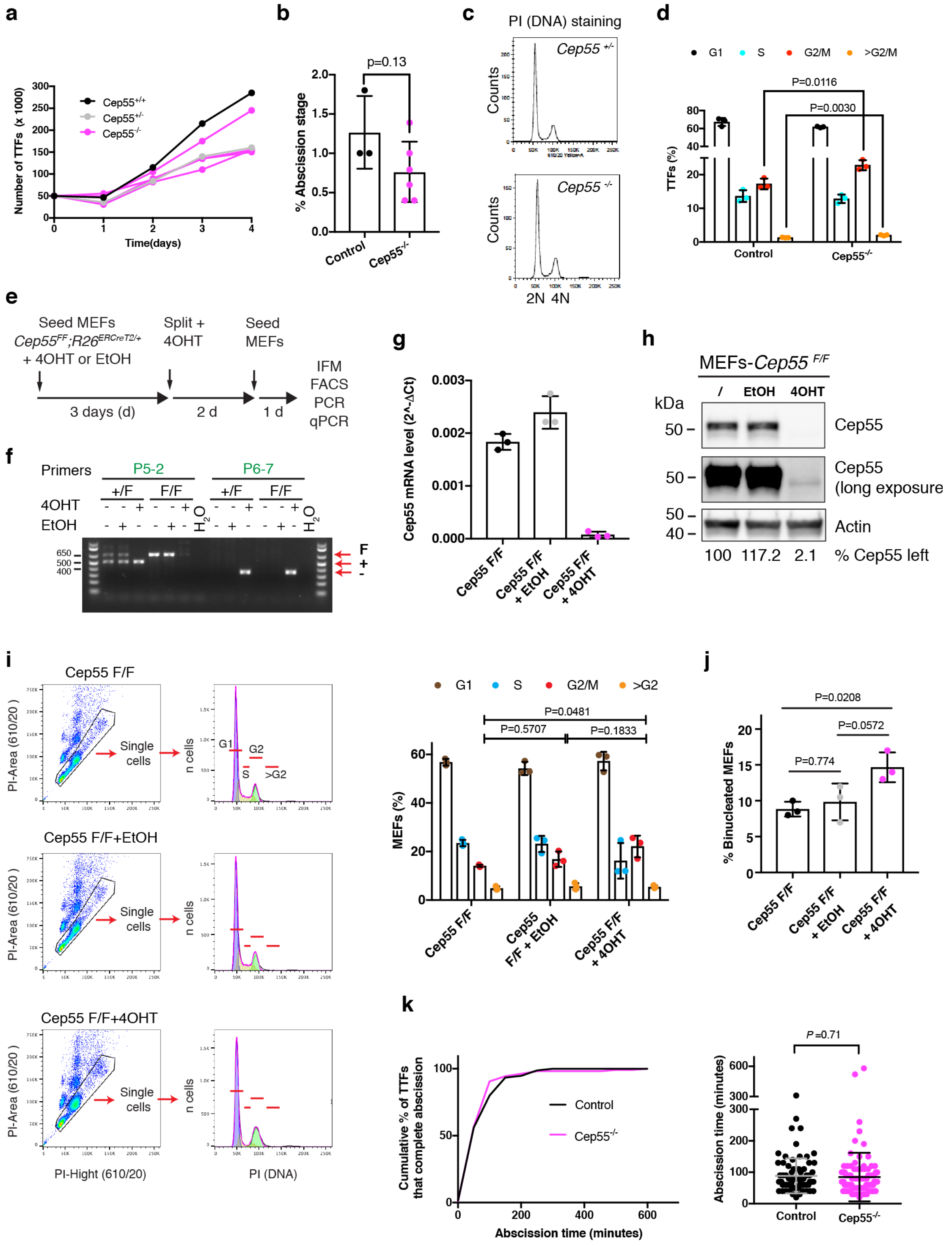

Supplementary Figure 11: **Cep55 is largely dispensable for division of primary fibroblasts.** (**a-d**) TTFs were cultured as indicated in Fig. 5d. (**a**) Cell counts at the indicated time points. n = 1 *Cep55*$^{+/+}$, 1 *Cep55*$^{+/-}$, and 4 *Cep55*$^{-/-}$ mice. (**b**) TTFs from Fig. 5e were analysed by IFM for cells at the abscission stage. n=1500 and 3000 cells from 3 and 6 experiments respectively. Bar charts show mean ± SD. (**c**) Representative FACS profiles after

DNA staining with propidium iodide (PI). (**d**) Summary of cell cycle distributions obtained by FACS analysis as in (d). Bar charts show mean ± SD. n = 3 mice per condition. >2500 cells quantified per condition. (**e**) Scheme for *Cep55* deletion in *Cep55*$^{F/F}$*; R26*$^{CreERT2/+}$ (F/F) MEFs by 4OHT, as used in (g-j). (**f**) PCR analysis of deletion of *Cep55* conditional (F) allele with the indicated primers (primer details in Fig. 1a). *Cep55*$^{+/F}$*; R26*$^{CreERT2/+}$ (+/F) MEFs were used as control. (**g**) qRT–PCR of Cep55 mRNA levels after the indicated treatments and collected as in (e). Results were normalized to Gapdh. Bar charts show mean ± SD. n = 3 independent experiments. (**h**) Western blots of protein extracts from day 6 with the indicated antibodies; n=1 experiment. (**i**) Gating strategy (left) and summary (right) of cell cycle distribution obtained by FACS analysis of MEFs of the indicated genotypes from (e) after DNA staining with PI. Bar charts show mean ± SD. n = 3 mice per condition; >30000 cells quantified per condition. P values for >G2 are P=0.60 for F/F vs F/F+EtOH, P=0.76 for F/F vs F/F+4OHT, P=0.96 for F/F+EtOH vs F/F+4OHT. (**j**) MEFs from (e) were stained for DNA (DAPI) and α-tubulin and analysed by IFM for binucleated cells. n = 3 mice per condition; 600 cells counted per condition. Bar charts show mean ± SD. (**k**) Distribution of the abscission times (left) and mean abscission times ± SD (right) in control (n=75 cells, 3 independent experiments) and *Cep55*$^{-/-}$ TTFs (n=106 cells, 3 independent experiments). *P* values were calculated using Student's two-tailed unpaired t test in b, d, j and k, and by one way ANOVA followed by Tukey's multiple comparisons test in i and j.

Supplementary Figure 12

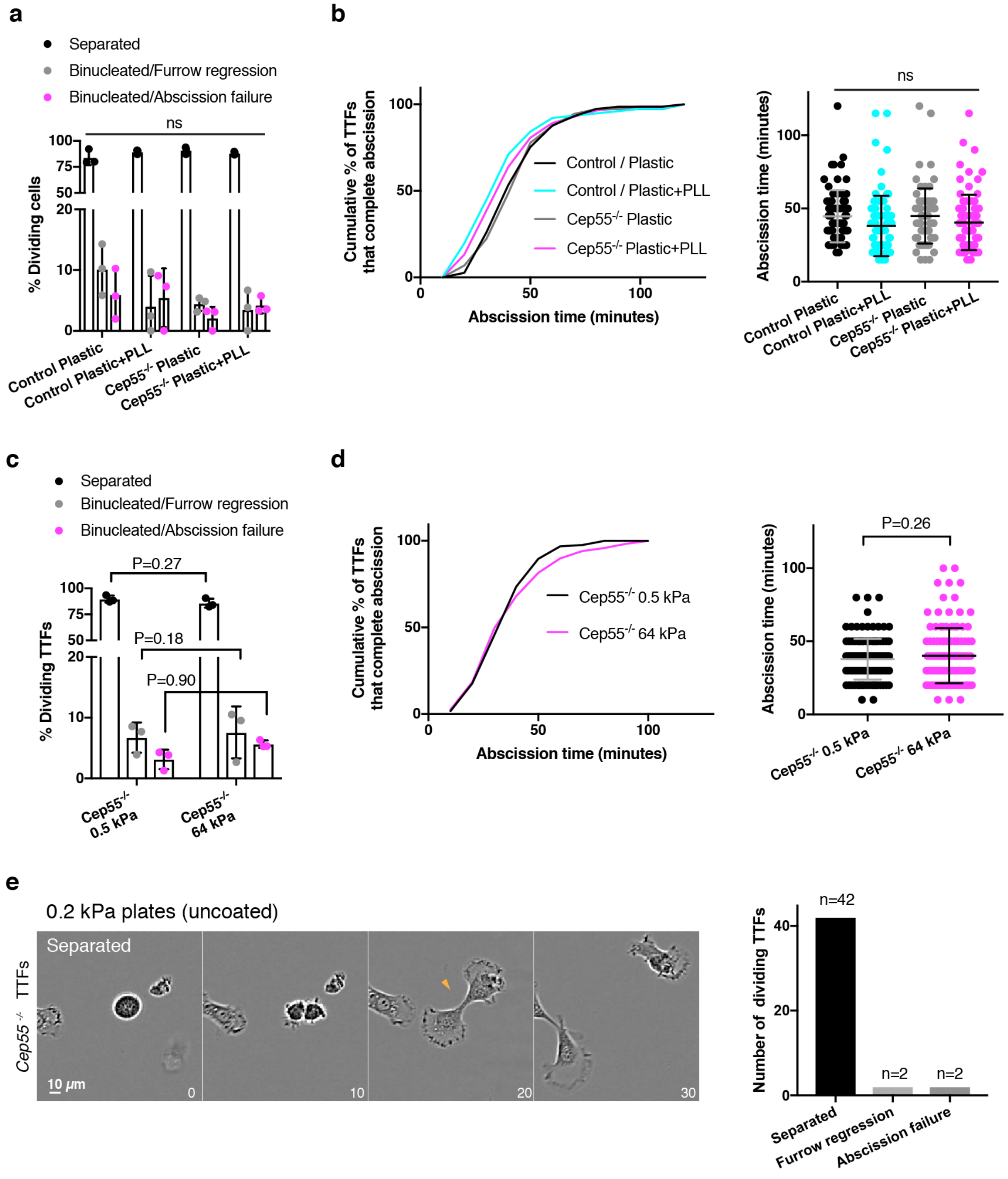

Supplementary Figure 12: ***Cep55* knockout fibroblasts complete abscission when cultured on PLL or on soft substrates.** (**a** and **b**) TTFs of the indicated genotypes were cultured either on plastic dishes (plastic) or plastic dishes coated with poly-l-lysine (Plastic+ PLL) and imaged as in Fig. 5d. (a) Quantification of dividing TTFs as defined in Fig. 5i. n = 3 mice per genotype; 125, 105, 131 and 110 cells quantified respectively. Bar charts show mean ± SD. (b) *P* values in (a) and (b) were calculated using two-way ANOVA, each mean compared to control (plastic), followed by Dunnett's multiple comparisons test and they are all non-significant (ns). For furrow regression, P values are 0.18, 0.22 and 0.14, respectively. For

abscission failure, P values are 0.99, 0.41 and 0.86, respectively. (b) Distribution of the abscission times (left) and mean abscission times ± SD (right). 72, 83, 70 and 63 cells per condition were analysed. P values calculated as in (a) are: 0.95, 0.99 and 0.98, respectively. (**c** and **d**) *Cep55*$^{-/-}$ TTFs were cultured either on soft (0.5 kPa) or stiff (64 kPa) plates coated with fibronectin and imaged as in Fig. 5d. (c) Quantification of dividing TTFs as defined in Fig.5i. n = 3 mice per genotype; 225 and 195 cells quantified respectively. Bar charts show mean ± SD. P value calculated with Student's two-tailed unpaired t test. (d) Distribution of the abscission times (left) and mean abscission times ± SD (right) (*P* values calculated using Student's two-tailed unpaired t test.) in *Cep55*$^{-/-}$ TTFs on 0.5 kPa plates (n=125 cells, 3 independent experiments) and 64 kPa plates (n=119 cells, 3 independent experiments). (**e**) *Cep55*$^{-/-}$ TTFs were cultured on soft (0.2 kPa) uncoated plates and classified as above (2 independent experiments).

Supplementary Figure 13

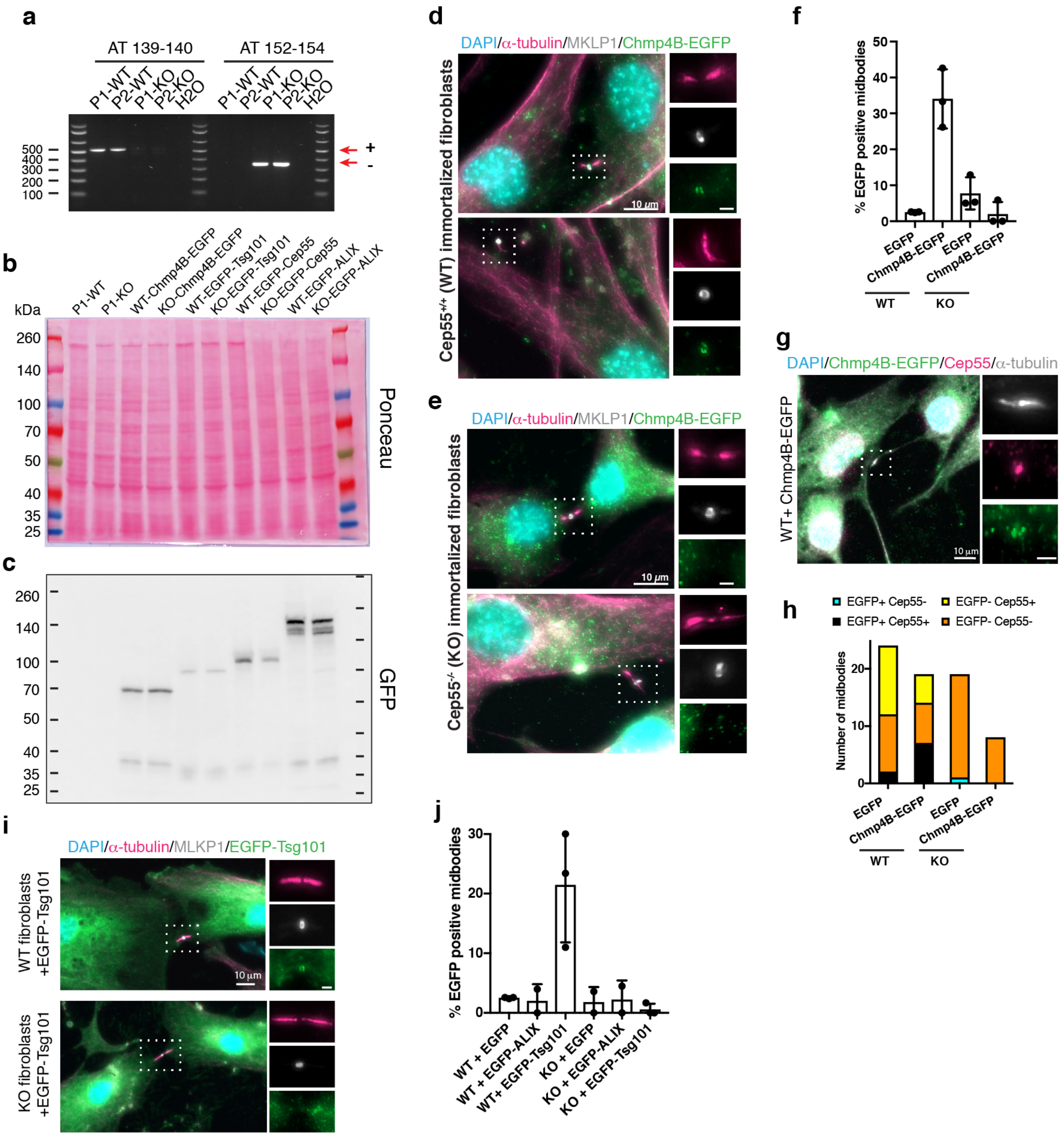

Supplementary Figure 13: **Characterization of the ESCRT pathway at the midbodies of fibroblasts.** (**a**) PCR analysis of two *Cep55*$^{+/+}$ (WT) and two *Cep55*$^{-/-}$ (KO) immortalized fibroblast lines with the indicated primers (primer details in Figure 1a). P1 and P2, parental lines 1 and 2, respectively; n=1 experiment. (**b** and **c**) Extracts from immortalized fibroblasts stably expressing the indicated proteins were stained with Ponceau (b) and for GFP (c); n= 2 independent experiments. (**d** and **e**). Immortalized fibroblasts of the indicated genotypes were stained with DAPI (DNA) and antibodies against α-tubulin, MKLP1 and GFP. Boxed regions are magnified at right. Scale bar in magnified regions, 2 μm. (**f**) Quantification of midbodies positive for EGFP in immortalized fibroblasts of the indicated genotypes from immunofluorescence images in (**d** and **e**). n = 3 independent experiments; 117, 156, 109 and 88 cells quantified per respective condition. Bar charts show mean ± SD. (**g**) *Cep55*$^{+/+}$ (WT) immortalized fibroblasts stably expressing Chmp4B-EGFP were stained with DAPI and antibodies against GFP, Cep55 and α-tubulin. An example of a midbody where Cep55 and

Chmp4B co-localize is shown. Boxed area is magnified at right. Scale bar in magnified regions, 2 μm. (**h**) Quantification of midbodies positive (+) and negative (-) for the indicated proteins from (g). (**i**) Immortalized fibroblasts stably expressing EGFP-Tsg101 were stained with DAPI and antibodies against α-tubulin, MKLP1 and GFP. Boxed areas are magnified at right. Scale bar in magnified regions, 2 μm. (**j**) Quantification of midbodies positive for EGFP (EGFP+) in immortalized fibroblasts harbouring the indicated constructs from (b and i). Means ± SD are shown. n = 3 (Tsg101) or 2 (ALIX) independent experiments; 57, 101, 90, 71, 91 and 94 cells were quantified per respective condition.

Supplementary Figure 14

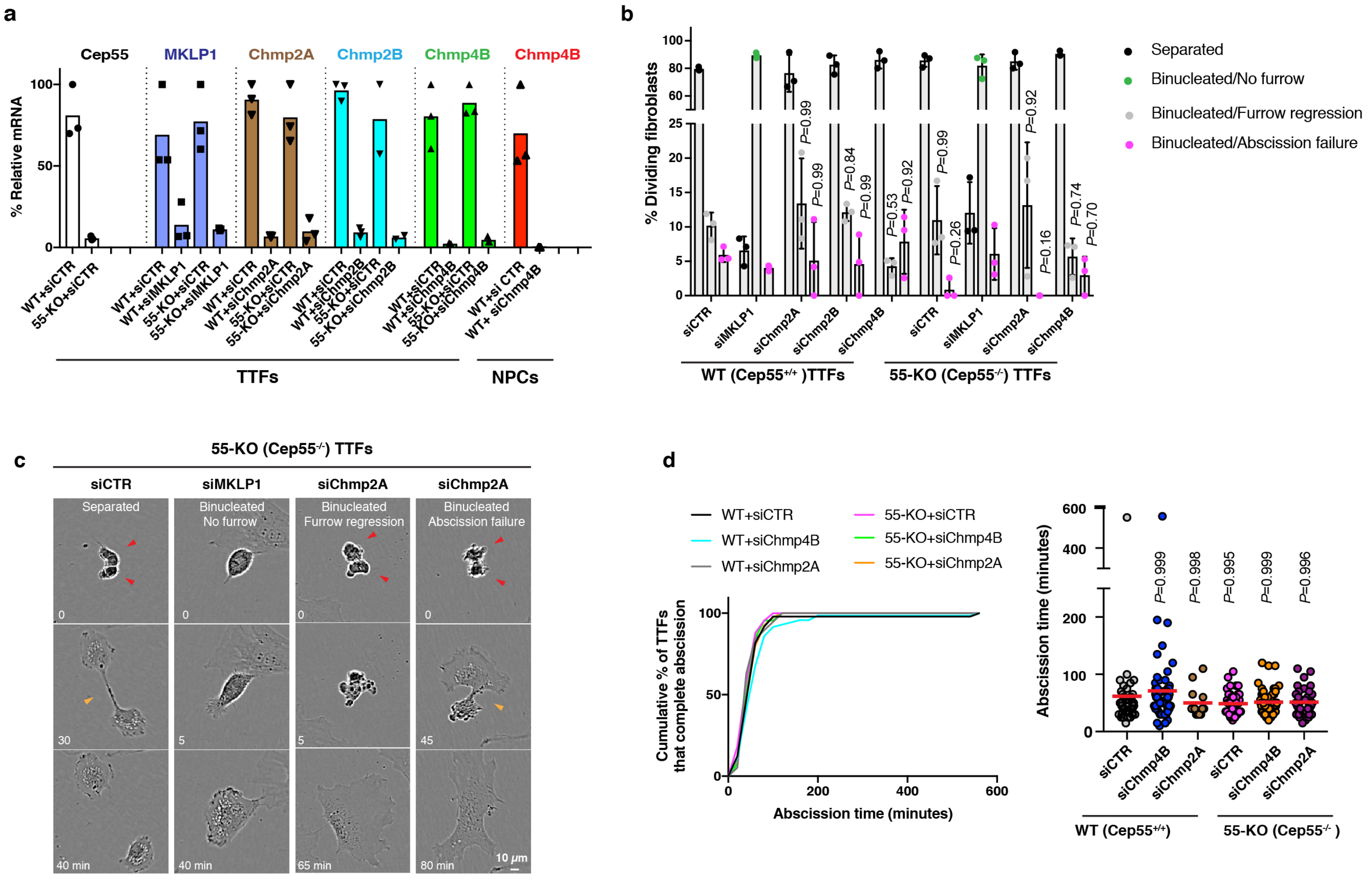

Supplementary Figure 14: **ESCRTs are dispensable for abscission in primary fibroblasts.** The indicated genes were knocked down and cells were followed by live-cell imaging as indicated in Fig. 7a. (**a**) qRT–PCR of relative mRNA levels of the indicated genes with specific siRNAs (si). Results were normalized to Gapdh, expressed as a percentage of the mRNA level in the "WT + CTR siRNA" condition. n = 3 independent experiments, except n=2 for KO+Chmp2B siRNA. CTR, control. Bars show mean value. (**b**) Quantification of live-cell imaging experiments as in (**c**). n = 3 independent experiments; 117, 75, 45, 123, 116, 122, 82, 47 and 107 cells were quantified per respective condition. Bars show mean ± SD. *P* values were calculated using two-way ANOVA, each mean compared to *Cep55*$^{+/+}$ + siCTR followed by Dunnett's multiple comparisons test, except for siChmp2B, for which the P value was calculated using one-way ANOVA. (**c**) Time-lapse images of *Cep55*$^{-/-}$ fibroblasts treated with the indicated siRNAs. Red arrowheads indicate daughter cells, while yellow arrowheads indicate the intercellular bridges. (**d**) Distribution of the abscission times (left) and mean abscission times ± SD (right) in cells of the indicated genotypes. Each dot represents one daughter cell doublet; means are shown as horizontal bars. n = 3 independent experiments; 42, 61, 13, 50, 67, and 40 cells were quantified per respective condition. *P* values were calculated using two-way ANOVA, each mean compared to *Cep55*$^{+/+}$ + siCTR followed by Dunnett's multiple comparisons test. In (a-d), *Cep55* KO indicates *Cep55*$^{tm1d/tm1d}$.

Supplementary Figure 15

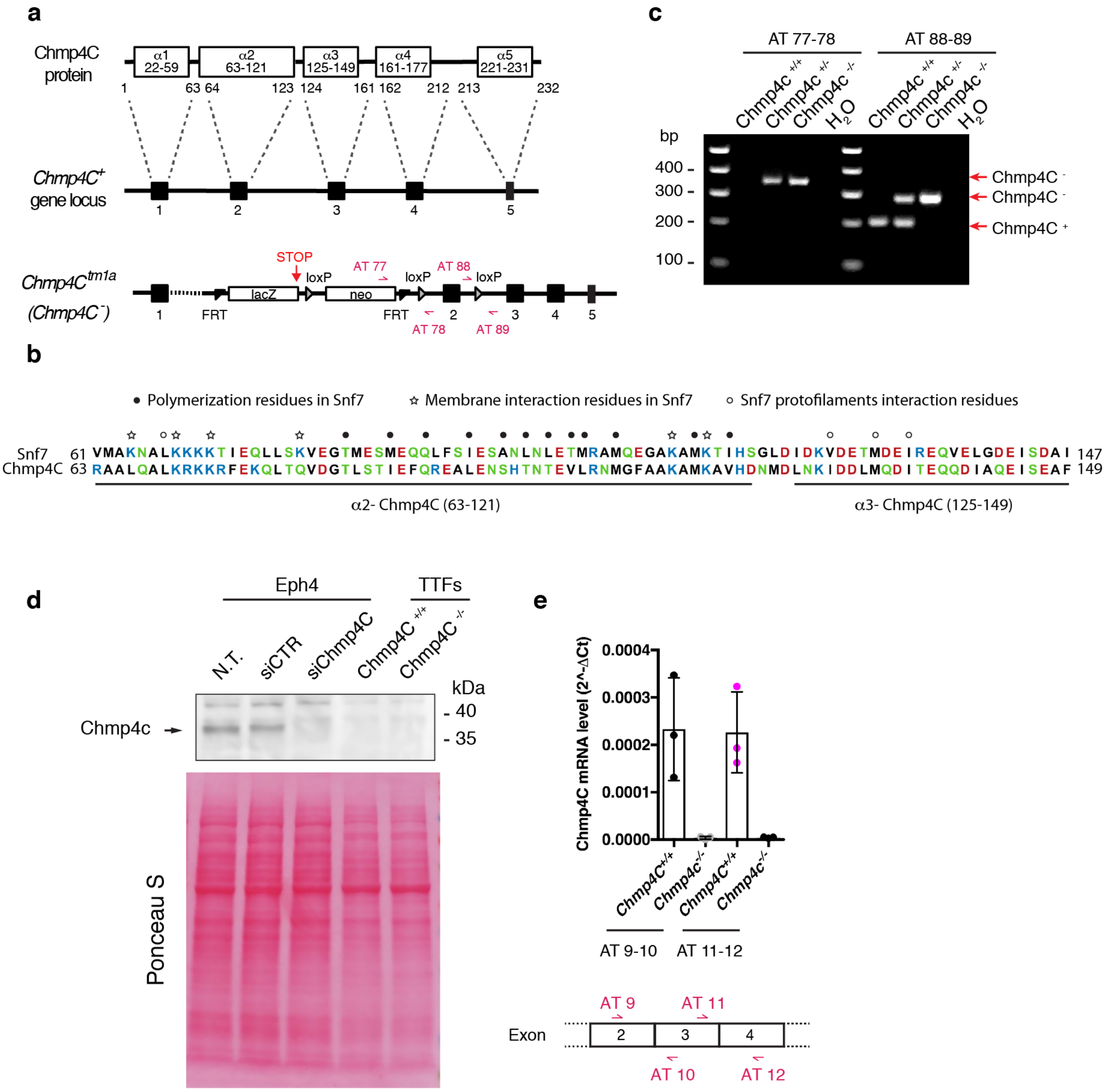

Supplementary Figure 15: **Validation of Chmp4C targeting strategy.** (**a**) Schematic representation of mouse Chmp4C secondary structure domains (α, alpha helix) and *Chmp4C* genomic locus, showing wild-type allele (+), the knockout first allele tm1a (including the selection cassette (neo), the LacZ trapping cassette, and LoxP and FRT recombination sites). Secondary structure domains were predicted as described in (Supplementary reference 1). (**b**) Saccharomyces cerevisiae Snf7 and mouse Chmp4C sequences were aligned using CLC sequence viewer. Amino acid residues with the same physicochemical properties are shown in the same font colour. Symbols on the top indicate critical residues for Snf7 functioning which were identified in (Supplementary reference 2). (**c**) PCR analysis of tail tip fibroblasts (TTFs) from newborn mice with primer pairs AT 77-78 and AT 88-89 shown in (a) to verify *Chmp4C* status; n= 3 independent experiments. (**d**) Western blots of protein extracts (30 µg per lane) from mouse immortalized epithelial cells (Eph4) and TTFs of the indicated genotypes. N.T., non-transfected; si, small interfering RNA; CTR, control; n= 2 independent experiments. (**e**) Quantitative reverse transcription PCR (qRT–PCR) of relative Chmp4c mRNA levels from TTFs of the indicated genotypes. Results were normalized to Gapdh. Means ± SD are shown (n = 3 independent experimental repeats for each condition). Schematic of the positions of primer pairs used in the qRT-PCR relative to Chmp4C exons is shown below the histogram.

Supplementary Figure 16

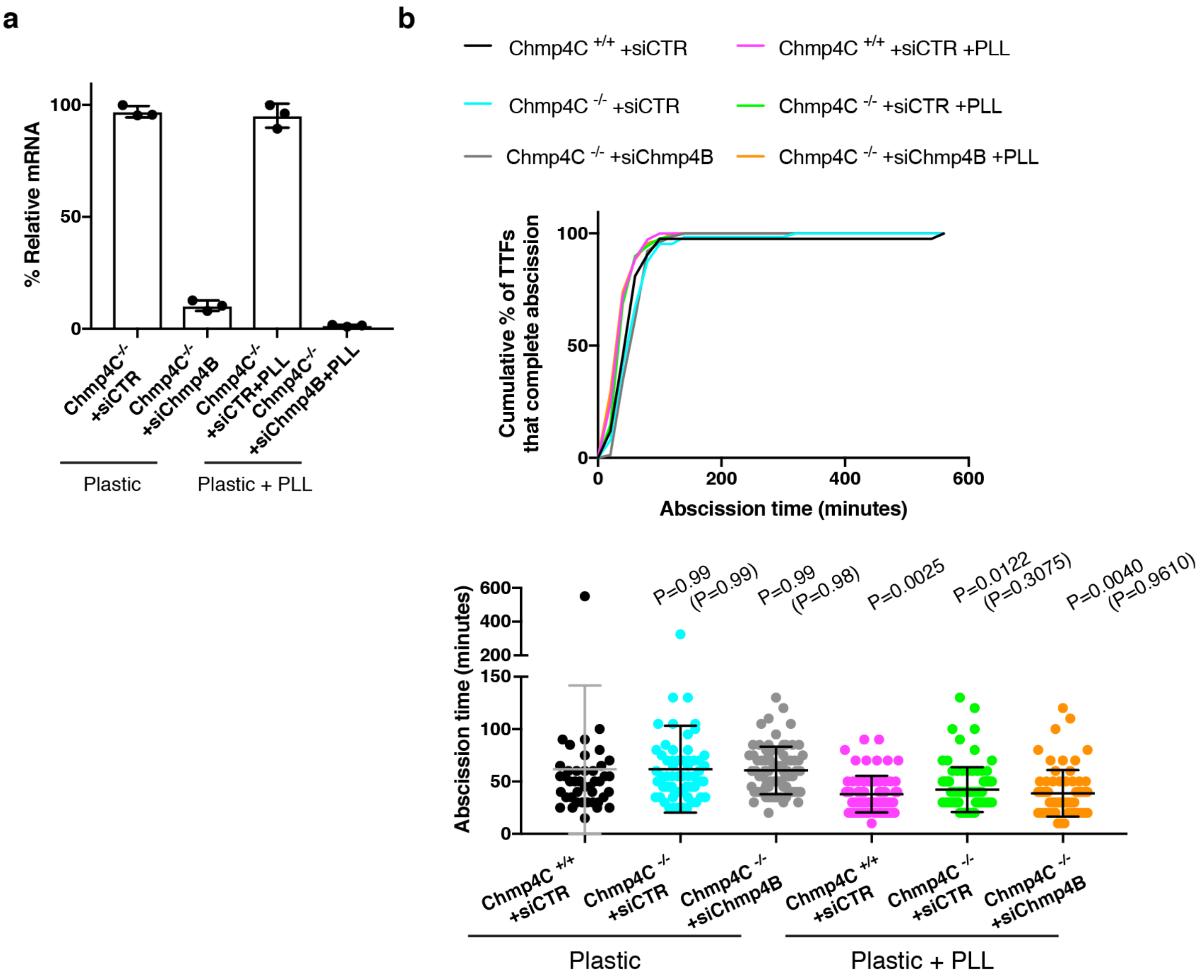

Supplementary Figure 16: **Chmp4B-and-C co-depleted fibroblasts efficiently complete abscission on poly-l-lysine coated dishes.** *Chmp4B* was knocked down in TTFs as indicated in Fig.7a. (**a**) qRT–PCR of relative mRNA levels of the indicated genes. Results were normalized to Gapdh, expressed as a percentage of the mRNA level in siCTR per each condition. n = 3 independent experiments. Bars show mean value ± SD. (**b**) Distribution of the abscission times (top) and mean abscission times ± SD (bottom) in cells of the indicated genotypes and treatments. Each dot represents one daughter cell doublet; means are shown as horizontal bars. n = 3 independent experiments; 42, 67, 40, 71, 89 and 69 cells were quantified per respective condition. *P* values were calculated using two-way ANOVA, each mean compared to *Cep55*$^{+/+}$ + siCTR on plastic, followed by Dunnett's multiple comparisons test. *P* values in brackets were calculated using one-way ANOVA for the two separate groups, plastic and plastic + PLL, each mean compared to *Cep55*$^{+/+}$ + siCTR, followed by Dunnett's multiple comparisons test.

Supplementary Figure 17

The following primers were used for PCR genotype (see Fig. 1a and Supplementary Figure 15a for their genomic positions):

P1: GGGATCGGCCATTGAACAAG
P 2: CTGCCAGTCTTTGCGTTTTCATTC
P 3: GATAAGCCTGACATTTCCCAACA
P 4: ACTGGTACCCAGGTCAGCTCTGG
P 5: TGTTGTGTTCCTATTTCTAGG
P 6: CTGGAGGGAATGAGCACTTTGAGA
P 7: CACCGAGCTATGTAGTTCTTTA
AT 77: GGGATCGGCCATTGAACAAG
AT 78: TTGGGAGAAAGCATCAGTTACA
AT 88: GTGTGGTGATTTTGGTAGGACTGG
AT 89: CCTTCGCTGGTGTTGGCATTGT

The following primers (forward/reverse) were used for Transnetyx genotype:

Cep55-tm1d: GTGGTTTGTCCAAACTCATCAATGT/ CCGCCTACTGCGACTATAGAGATAT
Cep55-WT: TGTGTCAGAAAGTAAAATCACATCATGTCT/ TGGCTTGTGTCCTCTTTTAATCTCA
Cep55-tm1c: CCTTTTTTTAATATATATACATATGTGTCAGAAAGTAAAATCACA/ GAACTGATGGCGAGCTCAGA
Cre: TTAATCCATATTGGCAGAACGAAAACG/ CAGGCTAAGTGCCTTCTCTACA
LacZ (Cep55-tm1a and Chmp4C-tm1a): CGATCGTAATCACCCGAGTGT/ CCGTGGCCTGACTCATTCC
Chmp4c-WT: GCCCGCCTTTAAGTAAGTCACT/ GTTGTCAAACCTAGCCTTGTTTTCT

The following primers (5' Oligo/ 3' Oligo) were used for subclonig ESCRTs components in the pBabe vector:

Chmp4B: GAAGATCtGCCACCATGTCGGTGTTCGGGAAGCTG/ CCCTCGAGCGAGTCGACCATGGATCCG
Tsg101: GGGGATCCATGGCGGTGTCCGAGAGTC/ GTGCGGCCGCTCAGTAGAGGTCACTAAGGCCCG
Alix: GACTCGAGATGGCGTCGTTCATCTGGGTGC/ GTGCGGCCGCTTACTGCTGTGGATAGTAGGACTGCTG

The following primers (forward/reverse) were used for qRT-PCR analysis:

Cep55:
qP 1/2: AAGGCAGAAGCAGACTCTTGGAGA/GTGGCGGACAGCTGGTTTTTCA
qP 3/4: TCGAGCTGGAAAAGAGAACAG/TGCTTCTCCACTTGAAGATAACC
qP 5/6: CTCAGCGAAAGGCAGACATCCAG/AGAATTCGGACCTGGGATAAGAGC
Gapdh: ACCCAGAAGACTGTGGATGG/ACACATTGGGGGTAGGAACA
MKLP1: TGTGACTGAACCCAAACCTGAGAA/GCTGCTGCAAGAGTTAGAG
Chmp4c: AT 9/10: CGCTGGAGAACTCGCACACC/CAATGTCCTGCTGCTCTGTGATA
AT 11/12: ATATCACAGAGCAGCAGGACATTG/GGGACGAGGACGGCACATTTG
Chmp2A: AGACGCCAGAGGAACTACTTC/ACCAGGTCTTTTGCCATGATTC
Chmp4b: GGAGAAGAGTTCGACGAGGAT/TGGTAGAGGGACTGTTTCGGG
Chmp2b: GGACCGAGCAGCCTTAGAG/CCAATCTTGGCCATCTTCTT

Supplementary references

1. Gatta, A.T. & Carlton, J.G. The ESCRT-machinery: closing holes and expanding roles. *Curr Opin Cell Biol* **59**, 121-132 (2019).
2. Tang, S. *et al.* Structural basis for activation, assembly and membrane binding of ESCRT-III Snf7 filaments. *Elife* **4** (2015).
