# Supplementary material for: Cep55 promotes cytokinesis of neural progenitors but is dispensable for most mammalian cell divisions
Source: Nat Commun. 2020 Apr 8;11:1746. doi: 10.1038/s41467-020-15359-w (PMC7142149; doi:10.1038/s41467-020-15359-w)
Supplement: Supplementary file 3 — Description of Additional Supplementary Files [file 41467_2020_15359_MOESM3_ESM.pdf]

Description of Additional Supplementary Files

File Name: Supplementary Movie 1
Description: . A Cep55+/+ mouse (right) and a Cep55-/- mouse (left) from the same litter at 9 days of age, showing the characteristic differences in body size and mobility between the two genotypes.

File Name: Supplementary Movie 2
Description: Bright-field movie of control neural progenitor cells (NPCs; Cep55F/F; R26CreERT2/+ + ethanol; see also Fig. 5g, top row). The first arrow indicates the cell to follow. The second arrow indicates the intercellular bridge before abscission.

File Name: Supplementary Movie 3
Description: Bright-field movie of Cep55 knockout NPCs (Cep55F/F; R26CreERT2/+ + 4-OHT; see also Fig. 5g, bottom row). The first arrow indicates the cell to follow. Note that the cell fails to complete abscission. The second and third arrows indicate the attempt of the binucleated cells to divide.

File Name: Supplementary Movie 4
Description: Phase contrast movie of Cep55+/+ primary mouse tail tip fibroblasts (TTFs; see also Fig. 5i, top row). The arrow indicates the intercellular bridge.

File Name: Supplementary Movie 5
Description: Phase contrast movie of Cep55-/- primary mouse TTFs (see also Fig. 5i, bottom row). The arrow indicates the intercellular bridge.

File Name: Supplementary Movie 6
Description: Phase contrast movie of Cep55-/- primary mouse TTFs cultured on poly-l-lysine (PLL; compare with bottom row in Fig. 5i, cultured without PLL). Arrows indicate the dividing cells to follow.

File Name: Supplementary Movie 7
Description: Live cell imaging of Cep55+/+ immortalized TTFs expressing Chmp4B-EGFP (green) and labelled with Sir-Tubulin (magenta) to visualize the microtubules. Arrow indicates Chmp4B-EGFP localization at the intercellular bridge before abscission (see also top row in Fig. 6m).

File Name: Supplementary Movie 8
Description: Live cell imaging of Cep55+/+ immortalized TTFs expressing Chmp4B-EGFP (green) and labelled with Sir-Tubulin (magenta) to visualize the microtubules. The first arrow indicates the intercellular bridge before abscission. The second arrow indicates Chmp4B-EGFP localization at the intercellular bridge after abscission has occurred (see also middle row in Fig. 6m).

File Name: Supplementary Movie 9
Description: Live cell imaging of Cep55-/- immortalized TTFs expressing Chmp4B-EGFP (green) and labelled with Sir-Tubulin (magenta) to visualize the microtubules. The first arrow indicates the intercellular bridge before abscission, with no localization of Chmp4B-EGFP (see also bottom row in Fig. 6m).

File Name: Supplementary Movie 10
Description: Live cell imaging of Cep55+/+ immortalized TTFs, expressing Chmp4B-EGFP (green) and labelled with Sir-Tubulin (magenta) to visualize the microtubules, cultured on PLL (compare with top row in Fig. 6m, cultured without PLL). Arrow indicates Chmp4B-EGFP localization at the intercellular bridge before abscission.

File Name: Supplementary Movie 11
Description: Phase contrast movie of Chmp4c-/- primary mouse TTFs treated with pooled control siRNAs (see also Fig. 7a-d). The arrow indicates the intercellular bridge.

File Name: Supplementary Movie 12
Description: Phase contrast movie of Chmp4c-/- primary mouse TTFs treated with pooled siRNAs against Chmp4B (see also first column in Fig. 7c). The first arrow indicates the cell to follow. The second arrow indicates the intercellular bridge.

File Name: Supplementary Movie 13
Description: Phase contrast movie of Chmp4c-/- primary mouse TTFs treated with pooled siRNAs against Chmp4B (see also third column in Fig. 7c). The first arrow indicates the cell to follow. The second arrow indicates the intercellular bridge. Note that the cell fails to complete abscission.

File Name: Supplementary Movie 14
Description: Phase contrast movie of Chmp4c-/- primary mouse TTFs cultured on PLL and treated with pooled siRNAs against Chmp4B (see also fourth column in Fig. 7c). Arrows indicate the dividing cells to follow.

File Name: Supplementary Movie 15
Description: Bright-field movie of a wild-type NPC treated with pooled control siRNAs (see also Fig. 7e, upper panel). The arrows indicate the intercellular bridge.

File Name: Supplementary Movie 16
Description: Bright-field movie of a wild-type NPC treated with pooled siRNAs against Chmp4B (see also Fig. 7e, lower panel). Note that the cell fails to complete abscission.
